# Symptom-based scoring technique by machine learning to predict COVID-19: a validation study

**DOI:** 10.1186/s12879-023-08846-0

**Published:** 2023-12-12

**Authors:** Amelia Nur Vidyanti, Sekar Satiti, Atitya Fithri Khairani, Aditya Rifqi Fauzi, Muhammad Hardhantyo, Herdiantri Sufriyana, Emily Chia-Yu Su

**Affiliations:** 1 Department of Neurology, Faculty of Medicine, Public Health and Nursing, Universitas Gadjah Mada, Yogyakarta, 55281 Indonesia; 2 Department of Neurology, Dr. Sardjito General Hospital, Yogyakarta, 55281 Indonesia; 3 Center for Health Policy and Management, Faculty of Medicine, Public Health and Nursing, Universitas Gadjah Mada, Yogyakarta, 55281 Indonesia; 4 Faculty of Health Science, Respati University Yogyakarta, Yogyakarta, 55281 Indonesia; 5 Graduate Institute of Biomedical Informatics, College of Medical Science and Technology, Taipei Medical University, 250 Wu-Xing Street, Taipei, 11031 Taiwan; 6 Department of Medical Physiology, Faculty of Medicine, Universitas Nahdlatul Ulama Surabaya, Surabaya, 60237 Indonesia; 7 Clinical Big Data Research Center, Taipei Medical University Hospital, Taipei, 11031 Taiwan; 8 Research Center for Artificial Intelligence in Medicine, Taipei Medical University, Taipei, 11031 Taiwan

**Keywords:** COVID-19, Clinical prediction rules, Validation study, Machine learning, Hospital referral

## Abstract

**Background:**

Coronavirus disease 2019 (COVID-19) surges, such as that which occurred when omicron variants emerged, may overwhelm healthcare systems. To function properly, such systems should balance detection and workloads by improving referrals using simple yet precise and sensitive diagnostic predictions. A symptom-based scoring system was developed using machine learning for the general population, but no validation has occurred in healthcare settings. We aimed to validate a COVID-19 scoring system using self-reported symptoms, including loss of smell and taste as major indicators.

**Methods:**

A cross-sectional study was conducted to evaluate medical records of patients admitted to Dr. Sardjito Hospital, Yogyakarta, Indonesia, from March 2020 to December 2021. Outcomes were defined by a reverse-transcription polymerase chain reaction (RT-PCR). We compared the symptom-based scoring system, as the index test, with antigen tests, antibody tests, and clinical judgements by primary care physicians. To validate use of the index test to improve referral, we evaluated positive predictive value (PPV) and sensitivity.

**Results:**

After clinical judgement with a PPV of 61% (*n* = 327/530, 95% confidence interval [CI]: 60% to 62%), confirmation with the index test resulted in the highest PPV of 85% (*n* = 30/35, 95% CI: 83% to 87%) but the lowest sensitivity (*n* = 30/180, 17%, 95% CI: 15% to 19%). If this confirmation was defined by either positive predictive scoring or antigen tests, the PPV was 92% (*n* = 55/60, 95% CI: 90% to 94%). Meanwhile, the sensitivity was 88% (*n* = 55/62, 95% CI: 87% to 89%), which was higher than that when using only antigen tests (*n* = 29/41, 71%, 95% CI: 69% to 73%).

**Conclusions:**

The symptom-based COVID-19 predictive score was validated in healthcare settings for its precision and sensitivity. However, an impact study is needed to confirm if this can balance detection and workload for the next COVID-19 surge.

**Supplementary Information:**

The online version contains supplementary material available at 10.1186/s12879-023-08846-0.

## Background

Several countries did not adequately respond to the coronavirus disease 2019 (COVID-19) pandemic situation [1]. Healthcare systems were overwhelmed by case surges regardless of the disease severity, as demonstrated by the emergence of omicron variants [2]. Proper healthcare systems need to balance workloads. This can be achieved by improving the referral system using simple yet precise and sensitive diagnostic predictions. Even though COVID-19 produces flu-like symptoms, a screening test for the general population is not widely available in many countries, including Indonesia [3]. As a result, identifying the most accurate predictive signs of COVID-19 is critical. This identification may also aid in the development of guidelines for self-isolation and illness prevention [4], in order to reduce healthcare workloads during case surges. By machine learning (ML), an accurate, self-reported symptom scoring of COVID-19 was developed (with an area under the receiver operating characteristics curve [AUC] of 0.76, 95% confidence interval [CI]: 0.74 to 0.78) [5]. However, the model should be validated before use in healthcare settings.

Increasing workloads are reported to reduce the quality of medical care provided. The COVID-19 pandemic increased delays of live-saving interventions from arrival time to hospital for patients with heart attacks in Singapore, particularly those with ST-segment elevation myocardial infarctions (71.4% vs. 80.9% of < 90-min intervention; *p* = 0.042; *n* = 303) [6]. For patients in the emergency department (ED), that were not admitted to the hospital during the pandemic, revisits within 3 days and death within 30 days were respectively higher in patients with a fever (hazard ratio [HR]: 1.33, 95% CI: 1.05 to 1.69) and chest pain (HR: 5.40, 95% CI: 1.28 to 22.84) compared to those with the same conditions before the pandemic [7]. A referral center or a dedicated clinic for COVID-19 has been established in many hospitals to reduce the ED workload, but false-positive (FP) patients are exposed to risks of in-hospital/nosocomial transmission. The sensitivity of front-door screening with universal mask wearing was only 50% to 70% in outpatient departments, 30.7% to 43.1% in hospital wards, and 28.7% to 40.1% in EDs [8]. A simple screening method with fewer FPs could help avoid in-hospital transmission and reduce ED workloads without sacrificing sensitivity. Therefore, better safety can be expected for both the hospital and community.

Only 10.34% (*n* = 24/232) prediction models were externally validated among those for COVID-19 and only 14.22% (*n* = 33/232) among those prediction models were diagnostic in term of classifying COVID-19 versus not COVID-19 [9]. Most clinical prediction studies for COVID-19 developed models for predicting severe-case, high-resource utilization, or deaths in hospital settings [10, 11]. Both applied complex ML models that do not provide access for public use; thus, we could not independently validate these models. Use of computed tomographic scans [10] and some laboratory blood tests (e.g., troponin) [11] may also not be accessible in primary care, which does not fit our goal. Two of the neurological symptoms of COVID-19, which are anosmia and ageusia, can be used as a screening technique to identify positive people with mild symptoms who would be advised to self-isolate [12]. A large-scale cohort study of a general population in the United States (*n* = 168,293) and the United Kingdom (*n* = 2,450,569) used symptoms of anosmia and ageusia as screening techniques to detect COVID-19-positive individuals [5]. Another study developed symptom-based COVID-19 predictions in hospital settings (with an AUC of 0.736, 95% CI: 0.674 to 0.798) [13]. But, it did not include anosmia and ageusia, and was conducted by applying a case–control paradigm for validation, which exposes a high risk of bias to this previous study [14]. Meanwhile, the general population study [5] needs external validation, particularly in healthcare settings. However, there has been no external validation of the model with a low risk of bias.

A prediction model requires a population similar to that sampled for developing the model; otherwise, a validation study is needed to describe the suitability of model application. Similarities of populations may particularly be reflected by the probability of COVID-19-positive results. In the general population, COVID-19 positives were 26.28% (*n* = 726/2763) in a United States cohort and 41.26% (*n* = 6452/15,638) in a United Kingdom cohort based on self-reported results of a reverse-transcription polymerase chain reaction (RT-PCR) [5]. Meanwhile, COVID-19 positives were 10.32% (*n* = 172/1667) in a hospital study, particularly outpatient populations [13]. Hospital patients in a referral center and an ED may reflect different predictive performances due to different propensities of how patients come to those units. In this study, we aimed to validate a symptom-based COVID-19 prediction model previously developed using an ML algorithm.

## Methods

The prediction model risk of bias assessment tool (PROBAST) guidelines (see Additional file 1) were applied to validate the index test [14]. We also reported this study according to the transparent reporting of a multivariable prediction model for individual prognosis or diagnosis (TRIPOD) guidelines (see Additional file 2) [15]. Ethical clearance of this study was approved by the Institutional Review Board of the Faculty of Medicine, Public Health, and Nursing, Universitas Gadjah Mada/Dr. Sardjito Hospital (no. KE/FK/0098/EC/2021). Written informed consent was obtained from all patients.

### Study design

We conducted a cross-sectional study at Dr. Sardjito Hospital, Yogyakarta, Indonesia. Medical records were evaluated if patients were admitted to (1) the COVID-19 referral center from March 4, 2020 to May 2, 2021 and (2) the ED from February 4, 2021 to December 5, 2021. We excluded patients who were confirmed to have COVID-19 by a rapid antigen test or RT-PCR for preoperative purposes. All samples were randomly taken without stratifying the outcome.

### Outcome definition

The predicted outcome was being COVID-19 positive or negative based on the RT-PCR from nasopharyngeal and oropharyngeal swabs. Due to some discrepancies of RT-PCR performance using various sample collection sites [16, 17], we used samples from both sites (nasopharynx and oropharynx) with any of them showed positive would define COVID-19. It had to be conducted within the same 14-day period from the admission date, considering the duration of the detectable result, which was from the 3^rd^ to the 17^th^ day [18]. Symptoms theoretically last for 5 days within that period, yet the real-world situation may vary.

### Index test

The index test was a prediction model using self-reported symptoms from previous studies [5]. Briefly, a free smartphone application was employed for use by the general population to record the evolution of their symptoms, including results of an RT-PCR test from throat swabs (the COVID-19 status was not yet known to be positive or negative). The model development was data-driven by an ML algorithm, i.e., logistic regression, that collected data from the United States (*n* = 168,293) and the United Kingdom (*n* = 2,450,569). In a general population of both countries, the positive predictive value (PPV) was 58% to 71%, while the negative predictive value (NPV) was 75% to 89%.

A prediction model was successfully developed to assess whether a patient was COVID-19 positive or negative by integrating the symptoms of anosmia and ageusia (primary signs) with a persistent cough, fatigue, and loss of appetite, after adjusting for age and sex. These predictors were selected from 14 variables which we considered: sex (female/male), age (years), body-mass index (BMI; kg/m^2^), loss of smell and taste (no/yes), fatigue (no/yes), shortness of breath (no/yes), fever (no/yes), persistent cough (no/yes), diarrhea (no/yes), delirium (no/yes), no appetite (no/yes), abdominal pain (no/yes), chest pain (no/yes), and hoarseness (no/yes). The predicted outcome was being COVID-19 positive or negative based on the RT-PCR results as self-reported by study participants.

The final model computed a score (Eq. 1). With the exception of age, all of the other aforementioned predictors were 1 if a subject responded yes or was male, and 0 otherwise. After that, a score was computed as a probability (Eq. 2). If the probability exceeded the threshold (i.e., 0.5 by default), then a subject was predicted to be COVID-19 positive. Otherwise, a subject was predicted to be COVID-19 negative.

1 $$\mathrm{Score}=-1.32-\left(0.01\times \mathrm{age}\right)+\left(0.44\times \mathrm{sex}\right)+\left(1.75\times \mathrm{anosmia\, and \,ageusia}\right)+\left(0.31\times \mathrm{persistent \,cough}\right)+\left(0.49\times \mathrm{fatigue}\right)+(0.39\times \mathrm{loss\, of \,appetite})$$

2 $$\mathrm{Probability}=\mathrm{exp}\left(\mathrm{score}\right)/(1+\mathrm{exp}(\mathrm{score}))$$

### Comparators

To imply how the index test potentially reduces hospital workload during a subsequent COVID-19 surge, we need several comparators. The rapid antigen and antibody tests were respectively assigned as comparators 1 and 2. The samples for rapid antigen test were derived from nasopharynx and oropharynx. The samples for rapid antibody test were derived from serum. Rapid antibody was defined as positive if IgM showed positive result. In addition, considering its robustness and accessibility, comparator 1 was also combined with the index test.

Meanwhile, comparator 3 was the clinical judgment of the primary care physicians who had referred the patient to the hospital in this study. The clinical judgement relied on the 2021 decree issued by Indonesia's Ministry of Health regarding the Clinical Guidelines and Management of COVID-19 in Healthcare Services in Indonesia [19]. Hospital referrals were exclusively for patients exhibiting moderate to severe symptoms of suspected COVID-19 infection.

### Statistical analysis

To evaluate the diagnostic performance, we counted true positives (TPs), false negatives (FNs), FPs, and true negatives (TNs). An evaluation metric of interest was the PPV, which could be correctly estimated to imply how the index test potentially reduced the workload. A higher PPV implied greater potential to reduce the healthcare workload. In the referral center, all patients had already been screened by the clinical judgment of primary care physicians. It was possible to use the data to evaluate the PPV. However, the sensitivity might not have been well-estimated for future data in primary care. This is because estimates of FNs were unknown based on data in the referral center. Meanwhile, patients came to ED arbitrarily, which was assumed similar to those in primary healthcare facilities that referred patients to the hospital.

For completeness, we also computed the NPV, sensitivity, and specificity. Bootstrapping 30 times was applied to infer the 95% CI. To bolster applicability of this study in diverse times, we also computed the evaluation metrics without or with case surges. The surge period was determined by visual inspection of a smoothing line of cases per 7 days. For discerning COVID-19 from other similar conditions, we also compared the evaluation among subjects without or with each of symptoms in the index test, particularly specific ones, i.e., loss of smell, loss of taste, and cough. We included a symptom category (e.g., no cough) as a subgroup if both positives and negatives were available. Data analyses were conducted using R version 4.0.2, and the codes are publicly available (see Code Availability).

## Results

### Baseline characteristics

**Fig. 1:**
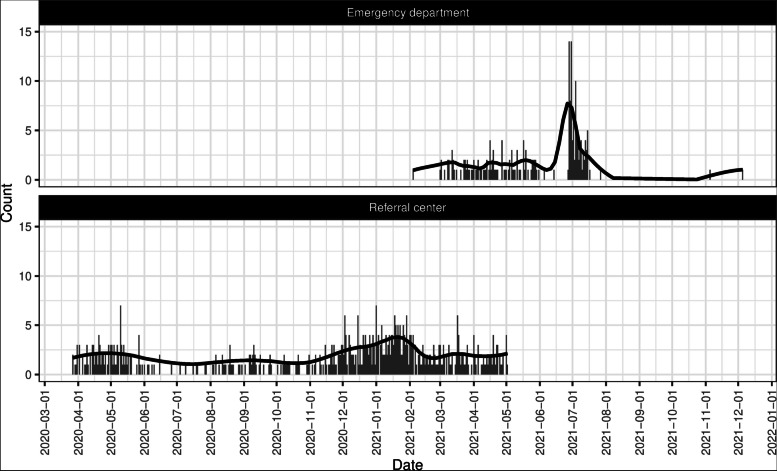
Count of samples per day the emergency department and referral center. The solid line shows the smoothing line per 7 days

We collected samples from the ED (*n* = 199) and referral center (*n* = 555). The data collection period and the daily count of samples are shown in Fig. 1. As represented by the samples, the number of patients in the ED surged during July 2021.

Only 9.55% (*n* = 19/199) of COVID-19 cases confirmed by RT-PCR test results were recorded in the ED (Table 1), probably due to the long waiting times. Accordingly, almost all of the results (*n* = 530/555, 95.40%) were recorded in the referral center. No difference in the positivity rate by the RT-PCR was found between the settings. More patients were tested with the antigen test in the ED compared to the referral center (*n* = 32/199, 16.08% vs. *n* = 48/555, 8.65%; *p* = 0.004). Contrarily, fewer patients were tested by the antibody test in the ED compared to the referral center (*n* = 5/199, 2.51% vs. *n* = 118/555, 21.26%; *p* < 0.001). There was no difference in the positivity by the antibody test, but it was higher by the antigen test in the referral center compared to that in the ED (*n* = 29/48, 60% vs. *n* = 2/32, 6%; *p* < 0.001).

**Table 1:** Baseline characteristics

Variable	Category – metric	Setting			
		Referral center (*n* = 555)	Emergency department (*n* = 199)	*p*-value^a^	
				Non-missing	Missing
**Outcome**					
COVID-19 by RT-PCR test	Negative –* n* (%)	203 (36.58)	9 (4.52)	> .05	< .001
	Positive –* n* (%)	327 (58.92)	10 (5.03)		
	Missing –* n* (%)	25 (4.50)	180 (90.45)		
**Index test**					
COVID-19 by symptom scoring ^b^	Negative –* n* (%)	263 (47.39)	197 (99.00)	> .05	< .001
	Positive –* n* (%)	35 (6.31)	0 (0.00)		
	Missing –* n* (%)	257 (46.30)	2 (1.00)		
**Comparator tests**					
COVID-19 by antigen test	Negative –* n* (%)	19 (3.42)	30 (15.08)	< .001	.004
	Positive –* n* (%)	29 (5.23)	2 (1.00)		
	Missing –* n* (%)	507 (91.35)	167 (83.92)		
COVID-19 by antibody test	Negative –* n* (%)	87 (15.68)	5 (2.51)	> .05	< .001
	Positive –* n* (%)	31 (5.58)	0 (0.00)		
	Missing –* n* (%)	437 (78.74)	194 (97.49)		
**Predictors**					
Age (years)	Non-missing – mean ± 95% CI	42.91 ± 1.74	50.25 ± 2.26	< .001	> .05
	Missing –* n* (%)	8 (1.44)	0 (0.00)		
Sex	Female –* n* (%)	279 (50.27)	90 (45.23)	> .05	> .05
	Male –* n* (%)	268 (48.29)	109 (54.77)		
	Missing –* n* (%)	8 (1.44)	0 (0.00)		
Loss of smell	No –* n* (%)	318 (57.30)	199 (100)	> .05	> .05
	Yes –* n* (%)	58 (10.45)	0 (0.00)		
	Missing –* n* (%)	179 (32.25)	0 (0.00)		
Loss of taste	No –* n* (%)	295 (53.15)	198 (99.50)	.031	> .05
	Yes –* n* (%)	14 (2.52)	1 (0.50)		
	Missing –* n* (%)	246 (44.33)	0 (0.00)		
Cough	No –* n* (%)	158 (28.47)	183 (91.96)	< .001	> .05
	Yes –* n* (%)	367 (66.13)	16 (8.04)		
	Missing –* n* (%)	30 (5.40)	0 (0.00)		
Fatigue	No –* n* (%)	212 (38.20)	139 (69.85)	.015	> .05
	Yes –* n* (%)	145 (26.13)	60 (30.15)		
	Missing –* n* (%)	198 (35.67)	0 (0.00)		
Skipped meals	No –* n* (%)	274 (49.37)	174 (87.44)	.048	< .001
	Yes –* n* (%)	61 (10.99)	23 (11.56)		
	Missing –* n* (%)	220 (39.64)	2 (1.00)		

^a^, Complete *p*-value indicates the statistical significance of the between-setting difference in data distribution using only non-missing values, while the missing *p*-value indicates the statistical significance of the between-setting difference in missing proportion; ^b^ only samples without missing values in any predictors under the default threshold (i.e., 0.5). *CI* Confidence interval, *NA* Not applicable, *RT-PCR* Reverse-transcription polymerase chain reaction

We compared symptoms in the referral center to those in the ED (Table 1). There was no difference in symptom checks between settings, except for skipped meals that were more likely checked in the ED than in the referral center (*n* = 197/199, 99.00% vs. *n* = 335/555, 60.36%; *p* < 0.001). Accordingly, symptom scoring in the ED was more likely to have been conducted in this study compared to that in the referral center (*n* = 197/199, 99.00% vs. *n* = 298/555, 53.70%; *p* < 0.001). The age distribution showed that patients were younger in the referral center than in the ED (42.91 ± 1.74 vs. 50.25 ± 2.26 years; *p* < 0.001). Meanwhile, higher positivity was identified for cough (*n* = 367/525, 69.90% vs. *n* = 16/199, 8.04%; *p* < 0.001), loss of taste (*n* = 14/309, 4.53% vs. *n* = 1/199, 0.50%; *p* = 0.031), fatigue (*n* = 145/357, 40.62% vs. *n* = 60/199, 30.15%; *p* = 0.015), and skipped meals (*n* = 61/335, 18.21% vs. *n* = 23/197, 11.68%; *p* = 0.048).

### Diagnostic performances

We validated the diagnostic performances by all tests by comparing them to RT-PCR results (Table 2). The index test was COVID-19 symptom score. For both settings, the comparators were antigen (comparator 1) and antibody (comparator 2) tests. Comparator 3 was a clinical judgment by the primary care physician, which was considered positive before the patient was admitted to the referral center. Due to missing values, only some evaluation metrics could be computed.

**Table 2:** Diagnostic performances

Setting	Test	Contingency (*n*)				Evaluation metric ± 95% CI (%^)^^a^			
		TPs	FNs	FPs	TNs	Sens	Spec ^b^	PPV	NPV ^b^
Emergency department	Index		10		9				48 ± 4
	Index or comparator 1	2			3				
	Comparator 1	2			3				
	Comparator 2								
Referral center	Index	30	150	5	105	17 ± 2	95 ± 1	85 ± 2	42 ± 2
	Index or comparator 1	55	7	5	2	88 ± 1	30 ± 6	92 ± 2	21 ± 3
	Comparator 1	29	12		6	71 ± 2			32 ± 3
	Comparator 2	24	33	6	50	39 ± 3	90 ± 2	79 ± 3	17 ± 2
	Comparator 3 ^c^	327		203				61 ± 1	

^a^ Interval estimates by 30 times bootstrapping; ^b^ patients with a negative outcome were likely underrepresented in the referral center; ^c^ under the assumption of positive COVID-19 by clinical judgment from primary care. *CI* Confidence interval, *FNs* False negatives, *FPs* False positives, *NPV* Negative predictive value, *PPV* Positive predictive value, *Sens*. Sensitivity, *Spec*. specificity, *TNs* True negatives, *TPs*, True positives

All patients with complete symptom checks and outcomes in the ED (*n* = 19) tested negative by the scoring, of which 48% (*n* = 9/19, 95% CI: 44% to 52%) were correctly negative (Table 2). This number was slightly lower in the referral center (*n* = 105/255, 42%, 95% CI: 40% to 44%); yet, patients with negative outcomes might not have been well-represented and were likely underrepresented. Meanwhile, patients with a positive score were more correctly positive (*n* = 30/35, 85%, 95% CI: 83% to 87%) than the clinical judgment from primary care physicians (*n* = 327/530, 61%, 95% CI: 60% to 62%). However, the index test achieved the lowest sensitivity (*n* = 30/180, 17%, 95% CI: 15% to 19%).

Furthermore, we evaluated a potential combination among the screening tests of interest. Patients with either positive scoring or antigen test in the referral center were more correctly positive (*n* = 55/60, 92%, 95% CI: 90% to 94%). Unlike symptom scoring only, more positives were detected (*n* = 55/62, 88%, 95% CI: 87% to 89%). Meanwhile, this number was also higher compared to that of the antigen test only (*n* = 29/41, 71%, 95% CI: 69% to 73%). Although this resulted in a lower specificity and NPV, the numbers were underestimated since negative outcomes were underrepresented in the referral center. The under-representativeness was implied by the lower NPV of the index test in the referral center (*n* = 105/455, 42%, 95% CI: 40% to 44%) compared to that in the ED (*n* = 9/19, 48%, 95% CI: 44% to 52%).

**Fig. 2:**
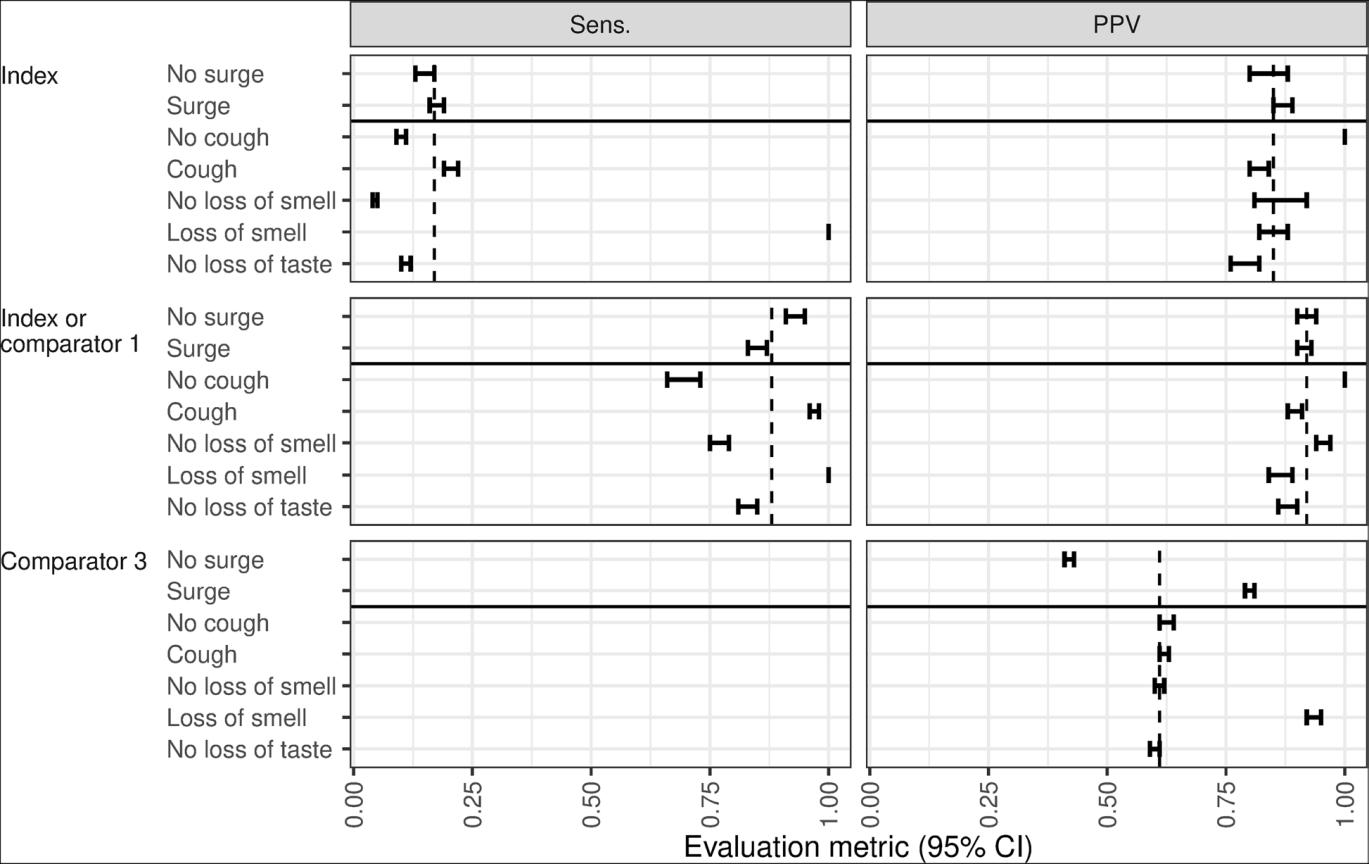
Diagnostic performances in referral center without or with case surges and different symptoms. The dashed line shows the average evaluation metric using all data. CI, confidence interval; PPV, positive predictive value; Sens., sensitivity

The combination of either positive scoring or antigen test in referral center was also evaluated for its applicability in diverse times and its ability in discriminating COVID-19 from other conditions with similar symptoms of loss of smell, loss of taste, and cough (Fig. 2). For simplicity, we only compared this combination with the index test and clinical judgment from primary care. To compute the evaluation metrics without or with case surges, we determined a surge period between November 1, 2020 and February 28, 2021 (Fig. 1). Patients with either positive scoring or antigen test in the referral center were most correctly positive (i.e., highest PPVs) and detecting most positives (i.e., highest sensitivities) without or with case surges. The same finding was also mostly found among patients without or with each of the selected symptoms, except: (1) indifferent PPV compared with the index test only among patients with no cough; (2) indifferent sensitivity compared with the index test among patients with loss of smell; and (3) slightly lower PPV compared with clinical judgment from primary care among patients with loss of smell.

## Discussion

### Summary of findings

COVID-19 positives by the index test were more correctly positive (PPV 85%, 95% CI: 83% to 87%) than those by clinical judgment. However, a COVID-19 negative should be re-evaluated by an antigen test. This demonstrated improvements in both the PPV (92%, 95% CI: 90% to 94%) and sensitivity (88%, 95% CI: 87% to 89%). The latter evaluation metric was higher than that of the antigen test only (71%, 95% CI: 69% to 73%). It was more likely to have been checked in the ED than in the referral center (*n* = 32/199, 16.08% vs. *n* = 48/555, 8.65%; *p* = 0.004), probably due to longer waiting times. This might have resulted in fewer patients being tested by the RT-PCR in the ED than in the referral center (*n* = 19/199, 9.55% vs. *n* = 530/555, 95.40%; *p* < 0.001). Therefore, COVID-19 negatives by the antigen test should also be re-evaluated with the index test to improve the sensitivity. This approach was also found more robust without or with case surges and each of the symptoms that may occur in other similar conditions, i.e., loss of smell, loss of taste, and cough.

### Comparison to previous studies

In the index test, both anosmia and ageusia were assigned the highest weight toward a COVID-19-positive result (Eq. 1). Combined with other predictors, this achieved PPVs of 58% to 71% in the development study [5] and 83% to 87% in this study. Notably, the latter PPV was achieved if the index test was applied when a patient was suspected of being COVID-19 positive by clinical judgment. Prediction models based on symptoms such as loss of smell and taste have been presented as useful methods for predicting COVID-19 diagnoses and as early indications of the success of containment efforts in future outbreaks [20]. Previous research on ML prediction models also reported loss of smell and taste as common symptoms that were closely related to COVID-19-positive results [20]. Similarly, a systematic review of 2757 patients found that those who reported loss of smell and taste had a six-fold greater chance of being positive for COVID-19, and those who suffered from anosmia and ageusia had a tenfold higher odds ratio (OR) [21].

The index test was developed using an ML algorithm [5], which is a data-driven instead of a knowledge-driven approach. New methods based on ML techniques have also been applied to investigate and predict olfactory impairment in different nasal illnesses [22]. As opposed to conventional statistical approaches, most modern ML methods rely on algorithms that may be used for modeling complicated interactions and correlations among several variables. Nonetheless, both conventional statistics and modern ML methods apply generalized additive modeling (e.g., linear/logistic regression).

In our study, the index test can be used as a starting point for determining whether a person is infected with COVID-19. A positive result based on the index test may predict with greater precision that the person is COVID-19 positive. However, a higher PPV may be a tradeoff with a lower NPV in the index test. This was implied by the NPV in the ED, which would have been similar to that of primary care had the index test been applied to make referral decisions. The propensity of a patient coming to primary care was assumed to be more similar to that of the ED than to that of the referral center.

Since a clinical judgment of COVID-19 positivity was made by primary care physicians before patients were referred to the hospital, higher positivity was identified for almost all of the symptoms evaluated in this study, compared to patients in the ED. More FPs, as demonstrated by the absolutely lower NPVs, resulted from neglecting the positives by the clinical judgment due to predicted negatives by either the index (*n* = 255) or antibody test (*n* = 83) in this study. Unfortunately, because of small sample sizes, we could not interpret the NPVs for neglected positives due to predicted negatives by either the combination of the index and antigen tests (*n* = 9) or the antibody test (*n* = 18) in the ED. Similarly, the lower NPV of the index test in the ED was also non-interpretable because of the small sample size (*n* = 19).

**Fig. 3:**
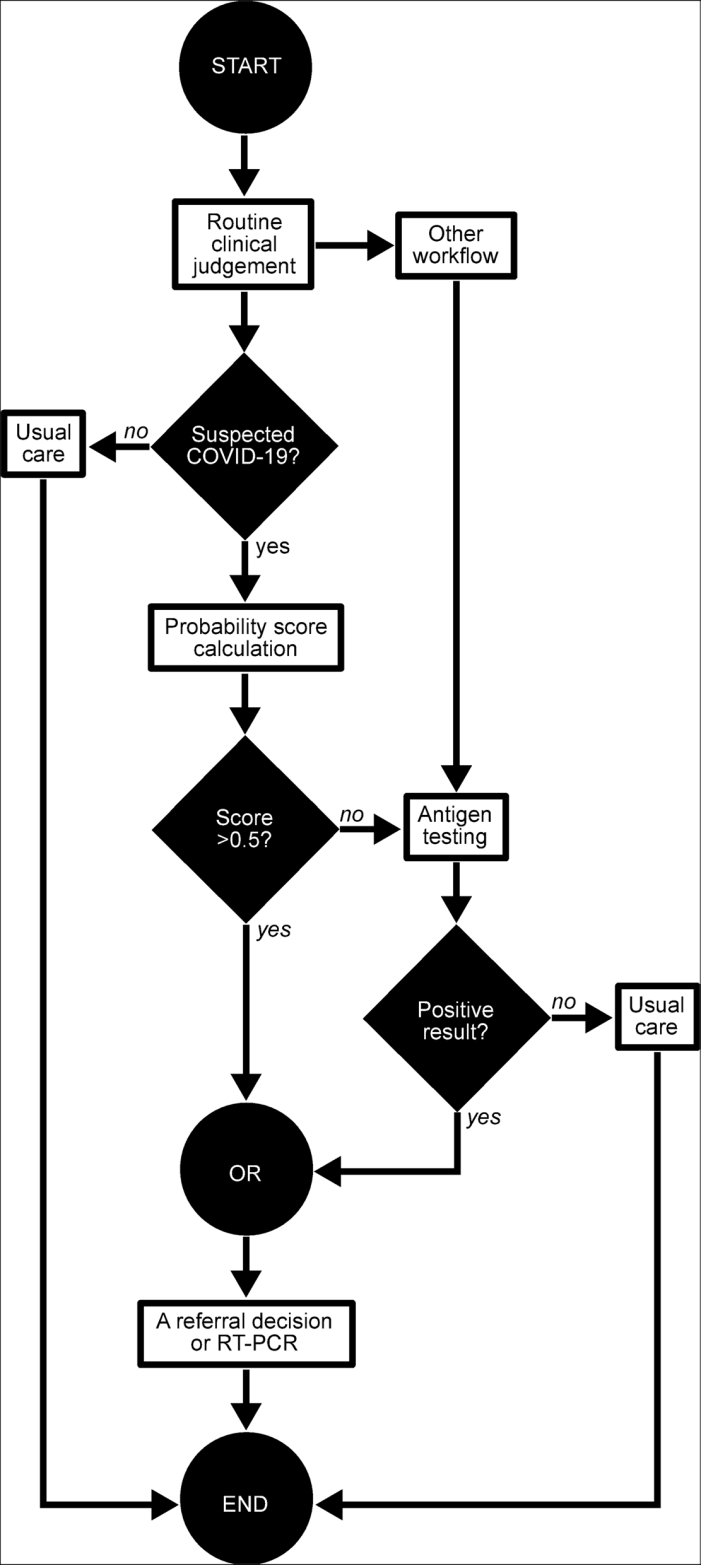
Proposed workflow of COVID-19 diagnoses during a case surge in primary care or the ED. COVID-19, coronavirus disease 2019; ED, emergency department; RT-PCR, reverse-transcription polymerase chain reaction

According to all of the findings, we proposed a screening workflow (Fig. 3). If a patient is considered COVID-19 positive by routine clinical judgment, then the probability score should be calculated by the index test; otherwise, usual care is applied. We provide a web application for a faster and more-precise calculation (https://predme.app/en/c19_predx/). If the score is evaluated to be COVID-19 positive, then the patient can be considered for a referral decision or an RT-PCR test; otherwise, an antigen test is conducted. A positive score does not require an antigen test. If the result of antigen test is already available, then a positive result by either the score or the antigen test should be considered for a referral decision or an RT-PCR test.

Notably, it is important to notice the importance of scoring instead of human judgement in final decision-making for a referral. Although the clinical judgement for COVID-19 diagnosis in this study was according to the standardized guidelines for COVID-19, the diagnostic performances were noisy (i.e., high variance), as shown in Fig. 2. A mechanical (objective) prediction, including scoring, is recommended over human (subjective) judgement, because it is vulnerable to cognitive bias [23]. In this study, the PPV of the clinical judgement without case surges were lower than that with case surges. The PPV of the clinical judgement was also lower in the absence of loss of smell which is the most notable symptom of COVID-19 during the pandemic. More precise and reliable diagnostic performances were demonstrated by the objective predictions by either positive scoring or antigen test. Therefore, a final decision-making for a referral should include such objective prediction, at least in addition to subjective human judgement. Nevertheless, a referral decision may also need another consideration, e.g., the severity level, depending on local policies.

### Strength and limitations

This study presents external validation of a prediction model developed by a large-scale study. Model development and validation used similar populations, but different countries and settings. Our validation data consisted of > 100 events in the referral center, which is recommended by the PROBAST guidelines.

However, we also identified limitations in this study. Our data collection period only covered the pre-omicron variants which more likely to have anosmia and ageusia than the omicron variant [24, 25]. Nonetheless, the index test also included other symptoms that are common among any variants and anosmia and ageusia are still diagnostically considered in the latest guideline for clinical management of COVID-19 from the World Health Organization published in August 2023 [26]. Furthermore, we found that the combination of either positive scoring or antigen test in referral center was robust among patients without or with loss of smell, loss of taste, or cough. The number of events in the ED was also insufficient, but we only evaluated metrics which were not considered if predicted positives (events) were true or false. We also did not recalibrate the threshold. Yet, the evaluation metric of interest, i.e., PPV, was already acceptable by combining with either the index test or antigen test. Finally, we excluded patients who underwent RT-PCR as the part of surgery preparation. Although they may be asymptomatic and would not perform the test unless they had surgery, they might show any subtle symptoms and the data from these subjects might be useful to develop a scoring system to diagnostically predict COVID-19 among mild cases. Nevertheless, the target population of the previous study, which developed the index test, did not resemble those who were underwent routine RT-PCR such as the part of surgery preparation. Generally, a lab testing which is not indicated by clinical judgement likely performs different to that among individuals which is indicated by clinical judgement, e.g., higher false positives. Therefore, as proposed in the workflow, the target population intended for the index test should be those after routine clinical judgement instead of routine RT-PCR testing.

## Conclusions

The precision of the symptom-based prediction model of COVID-19 has been validated according to the PPV. A diagnostic workflow is proposed by combining the model and antigen test. Therefore, we can expect a balanced tradeoff between robust detection and a balanced workload during the next COVID-19 surge, particularly in low-resource settings of which the RT-PCR is not readily accessible. We suggest an impact study for future investigations to evaluate the effect of model deployment on patient outcomes and healthcare workloads.

### Supplementary Information

**Additional file 1. **Prediction model risk of bias assessment tools (PROBAST).

**Additional file 2. **The transparent reporting of a multivariable prediction model for individual prognosis or diagnosis (TRIPOD).

## Data Availability

The datasets used and/or analyzed during the current study are available from the corresponding author on reasonable request. Analysis codes were shared in a public repository (https://github.com/herdiantrisufriyana/colab_scov2sar). Raw data are needed to reproduce the codes.

## Abbreviations

AUC: Area under the receiver operating characteristics curve
BMI: Body-mass index
CI: Confidence interval
COVID-19: Coronavirus disease 2019
ED: Emergency department
FN: False negative
FP: False positive
HR: Hazard ratio
ML: Machine learning
NPV: Negative predictive value
PPV: Positive predictive value
PROBAST: Prediction model risk of bias assessment tool
RT-PCR: Reverse-transcription polymerase chain reaction
TN: True negative
TP: True positive
TRIPOD: Transparent reporting of a multivariable prediction model for individual prognosis or diagnosis
