# Supplementary material for: Symptom-based scoring technique by machine learning to predict COVID-19: a validation study
Source: BMC Infect Dis. 2023 Dec 12;23:871. doi: 10.1186/s12879-023-08846-0 (PMC10716953; doi:10.1186/s12879-023-08846-0)
Supplement: Supplementary file 1 — Additional file 1. Prediction model risk of bias assessment tools (PROBAST). [file 12879_2023_8846_MOESM1_ESM.docx]

Prediction model risk of bias assessment tools (PROBAST).

| DOMAIN 1: Participants | | | | | |
| --- | --- | --- | --- | --- | --- |
| Risk of Bias | | | | | |
| *Describe the sources of data and criteria for participant selection:* | | | | | |
|  | • | We conducted a cross-sectional study at Dr. Sardjito Hospital, Yogyakarta, Indonesia. | | | |
|  | • | Medical records were evaluated if patients were admitted to (1) the COVID-19 referral center from March 4, 2020 to May 2, 2021 and (2) the ED from February 4, 2021 to December 5, 2021. | | | |
|  | • | We excluded patients who were confirmed to have COVID-19 by a rapid antigen test or RT-PCR for preoperative purposes. | | | |
|  | |  | | Dev | Val |
| 1.1 Were appropriate data sources used, e.g. cohort, RCT or nested case-control study data? | | | | - | Y |
| 1.2 Were all inclusions and exclusions of participants appropriate? | | | | - | Y |
| Risk of bias introduced by selection of participants | | | RISK: | - | Low |
|  |  |  | *(low/ high/ unclear)* |  |  |
| *Rationale of bias rating:* | | | | | |
|  | ❖ | This study validated a diagnostic prediction model which required a cross-sectional design. | | | |
|  | ❖ | The inclusion criteria were appropriate according to the intended use of the prediction model in this study. | | | |
|  | ❖ | The exclusion criteria were appropriate since these patients were pre-selected by universal screening instead of clinical judgment of patients’ complaints. | | | |

| DOMAIN 2: Predictors | | | | | |
| --- | --- | --- | --- | --- | --- |
| Risk of Bias | | | | | |
| *List and describe predictors included in the final model, e.g. definition and timing of assessment:* | | | | | |
|  | • | These predictors were selected from 14 variables which we considered: sex (female/male), age (years), body-mass index (BMI; kg/m2), loss of smell and taste (no/yes), fatigue (no/yes), shortness of breath (no/yes), fever (no/yes), persistent cough (no/yes), diarrhea (no/yes), delirium (no/yes), no appetite (no/yes), abdominal pain (no/yes), chest pain (no/yes), and hoarseness (no/yes). | | | |
|  | • | We conducted a cross-sectional study at Dr. Sardjito Hospital, Yogyakarta, Indonesia. Medical records were evaluated if patients were admitted to (1) the COVID-19 referral center from March 4, 2020 to May 2, 2021 and (2) the ED from February 4, 2021 to December 5, 2021. | | | |
|  | • | It had to be conducted within the same 14-day period from the admission date, considering the duration of the detectable result, which was from the 3rd to the 17th day. Symptoms theoretically last for 5 days within that period, yet the real-world situation may vary. | | | |
|  | |  | | Dev | Val |
| 2.1 Were predictors defined and assessed in a similar way for all participants? | | | | - | PY |
| 2.2 Were predictor assessments made without knowledge of outcome data? | | | | - | PY |
| 2.3 Are all predictors available at the time the model is intended to be used? | | | | - | PY |
| Risk of bias introduced by predictors or their assessment | | | RISK: | - | Low |
|  |  |  | *(low/ high/ unclear)* |  |  |
| *Rationale of bias rating:* | | | | | |
|  | ❖ | The predictors were simple to be assessed; thus, the assessment results were considerably similar. | | | |
|  | ❖ | Some of outcome knowledge were inevitably known by the assessors, because the patients may have the reference and comparator test results, particularly those in the referral center. But, the assessors were blind to the index test. | | | |
|  | ❖ | The predictors (mostly symptoms) were available within the time of prediction period in this study. | | | |

| DOMAIN 3: Outcome | | | | | |
| --- | --- | --- | --- | --- | --- |
| Risk of Bias | | | | | |
| *Describe the outcome, how it was defined and determined, and the time interval between predictor assessment and outcome determination:* | | | | | |
|  | • | The predicted outcome was being COVID-19 positive or negative based on the RT-PCR from nasopharyngeal and oropharyngeal swabs. It had to be conducted within the same 14-day period from the admission date, considering the duration of the detectable result, which was from the 3rd to the 17th day. | | | |
|  | • | A prediction model was successfully developed to assess whether a patient was COVID-19 positive or negative by integrating the symptoms of anosmia and ageusia (primary signs) with a persistent cough, fatigue, and loss of appetite, after adjusting for age and sex. | | | |
|  | • | It had to be conducted within the same 14-day period from the admission date, considering the duration of the detectable result, which was from the 3rd to the 17th day. Symptoms theoretically last for 5 days within that period, yet the real-world situation may vary. | | | |
|  | |  | | Dev | Val |
| 3.1  Was the outcome determined appropriately? | | | | - | Y |
| 3.2  Was a pre-specified or standard outcome definition used? | | | | - | Y |
| 3.3  Were predictors excluded from the outcome definition? | | | | - | Y |
| 3.4  Was the outcome defined and determined in a similar way for all participants? | | | | - | Y |
| 3.5  Was the outcome determined without knowledge of predictor information? | | | | - | PY |
| 3.6  Was the time interval between predictor assessment and outcome determination appropriate? | | | | - | PY |
| Risk of bias introduced by the outcome or its determination | | | RISK: | - | Low |
|  |  |  | *(low/ high/ unclear)* |  |  |
| *Rationale of bias rating:* | | | | | |
|  | ❖ | The outcome measurement was a gold standard diagnostic measurement of COVID-19. | | | |
|  | ❖ | Because this study used real-world data, the RT-PCR was likely decided according to the symptom, but the clinicians made decisions without knowing the index test. | | | |
|  | ❖ | Since this study developed a diagnostic prediction model, the predictors (mostly symptoms) were available within the time of prediction period in this study, which was the detectable period of the RT-PCR. | | | |

| DOMAIN 4: Analysis | | | | | |
| --- | --- | --- | --- | --- | --- |
| Risk of Bias | | | | | |
| *Describe numbers of participants, number of candidate predictors, outcome events and events per candidate predictor:* | | | | | |
|  | • | Table 1. Baseline characteristics  Negative – n (%) 203 (36.58)  Positive – n (%) 327 (58.92) | | | |
|  | • | These predictors were selected from 14 variables which we considered: sex (female/male), age (years), body-mass index (BMI; kg/m2), loss of smell and taste (no/yes), fatigue (no/yes), shortness of breath (no/yes), fever (no/yes), persistent cough (no/yes), diarrhea (no/yes), delirium (no/yes), no appetite (no/yes), abdominal pain (no/yes), chest pain (no/yes), and hoarseness (no/yes). | | | |
|  | • | (Events per candidate predictor are not applicable for a validation study.) | | | |
| *Describe how the model was developed (for example in regards to modelling technique (e.g. survival or logistic modelling), predictor selection, and risk group definition):* | | | | | |
|  | • | (Not applicable) | | | |
| *Describe whether and how the model was validated, either internally (e.g. bootstrapping, cross validation, random split sample) or externally (e.g. temporal validation, geographical validation, different setting, different type of participants):* | | | | | |
|  | • | (Not applicable) | | | |
| *Describe the performance measures of the model, e.g. (re)calibration, discrimination, (re)classification, net benefit, and whether they were adjusted for optimism:* | | | | | |
|  | • | To evaluate the diagnostic performance, we counted true positives (TPs), false negatives (FNs), FPs, and true negatives (TNs). An evaluation metric of interest was the PPV, which could be correctly estimated to imply how the index test potentially reduced the workload. A higher PPV implied greater potential to reduce the healthcare workload. In the referral center, all patients had already been screened by the clinical judgment of primary care physicians. It was possible to use the data to evaluate the PPV. But, the sensitivity might not have been well-estimated for future data in primary care. This is because estimates of FNs were unknown based on data in the referral center. Meanwhile, patients came to ED arbitrarily, which was assumed similar to those in primary healthcare facilities that referred patients to the hospital. | | | |
| *Describe any participants who were excluded from the analysis:* | | | | | |
|  | • | (There was no description of exclusion after data collection.) | | | |
| *Describe missing data on predictors and outcomes as well as methods used for missing data:* | | | | | |
|  | • | (There was no description for missing imputation.) | | | |
|  | |  | | Dev | Val |
| 4.1  Were there a reasonable number of participants with the outcome? | | | | - | Y |
| 4.2  Were continuous and categorical predictors handled appropriately? | | | | - | Y |
| 4.3  Were all enrolled participants included in the analysis? | | | | - | Y |
| 4.4  Were participants with missing data handled appropriately? | | | | - | PY |
| 4.5 Was selection of predictors based on univariable analysis avoided? | | | | - |  |
| 4.6  Were complexities in the data (e.g. censoring, competing risks, sampling of controls) accounted for appropriately? | | | | - | PY |
| 4.7  Were relevant model performance measures evaluated appropriately? | | | | - | Y |
| 4.8  Were model overfitting and optimism in model performance accounted for? | | | | - |  |
| 4.9 Do predictors and their assigned weights in the final model correspond to the results from multivariable analysis? | | | | - |  |
| Risk of bias introduced by the analysis | | | RISK: | - | Low |
|  |  |  | *(low/ high/ unclear)* |  |  |
| *Rationale of bias rating:* | | | | | |
|  | ❖ | The number of events were >100. | | | |
|  | ❖ | There was no categorization of numerical variables. | | | |
|  | ❖ | There was no exclusion after data collection. | | | |
|  | ❖ | No imputation and complexity handling was applied, but this validation design fits the real-world intended use of the model being evaluated. | | | |
|  | ❖ | We primarily inferred the conclusion based on PPV which could be correctly estimated from the data. | | | |

| Overall judgement about risk of bias of the prediction model evaluation | | |
| --- | --- | --- |
| Overall judgement of risk of bias | RISK: | Low |
|  | *(low/ high/ unclear)* |  |
| *Summary of sources of potential bias:* | | |
| This conclusion was only applied for the validation in the referral center, as explained in the main text. | | |
